# Bruits over Thoracic Aneurysm

**Published:** 1911-06-24

**Authors:** 


### Medicine.

### BRUITS OVER THORACIC ANEURYSM.

The presence or absence of a bruit is often spoken of as being of prime importance in connection with the diagnosis of aortic aneurysm. As a matter of fact, however, bruits are more often absent than present when the aneurysm affects the thoracic aorta so that the stethoscope cannot be applied directly to the sac. Even when there is a bruit associated with an aneurysm of the aortic arch there is considerable probability that this bruit is due not to the aneurysm itself, but to simultaneous atheromatous changes in the aortic valves; both aneurysm and aortic disease are secondary as a rule to atheroma, very often to syphilitic atheroma, and hence it happens that aortic systolic bruits or even aortic systolic and diastolic bruits, are present at the same time as an aneurysm without these bruits being produced actually within the aneurysmal sac. Indeed it would be surprising if a bruit were always present over an aneurysm, for murmurs are due to the production of abnormal eddies and whirls in the blood stream, particularly when there is a force driving the blood past an irregularity of some kind; if an aneurysm were to arise suddenly upon a normal vessel, for the time being there would be a bruit over it, but presently, if the aneurysmal sac remained of the same size all the while, blood clot would be deposited in those parts of the aneurysm in which back eddies and swirls were most marked, and in this way the interior of the sac would be made smooth once more, so that the blood stream would pass through it smoothly and without a continuance of that disturbance of the free onflow of the blood which is necessary for the production of a bruit. The presence of a bruit over an aneurysmal sac is therefore evidence of the absence of a deposition of clot within the sac or of there having been recently a change in the outline of the sac, generally as the result of additional dilatation. When an aneurysm has been approximately of the same size for weeks or months it is the rule rather than the exception for a bruit not to be heard over it unless the stethoscope can be pressed into one wall of it, altering its contour, as may be the case with an abdominal aneurysm. When the lesion is intrathoracic the absence of a bruit is very far from indicating absence of aneurysm.

